# North-south scientific collaborations on research datasets: a longitudinal analysis of the division of labor on genomic datasets (1992–2021)

**DOI:** 10.3389/fdata.2023.1054655

**Published:** 2023-06-15

**Authors:** Sarah Bratt, Mrudang Langalia, Abhishek Nanoti

**Affiliations:** ^1^School of Information (iSchool), University of Arizona, Tucson, AZ, United States; ^2^Eller College of Management, University of Arizona, Tucson, AZ, United States

**Keywords:** scientific collaboration, genomics, GenBank, research data, Sustainable Development Goal (SDGs)

## Abstract

Collaborations between scientists from the global north and global south (N-S collaborations) are a key driver of the “fourth paradigm of science” and have proven crucial to addressing global crises like COVID-19 and climate change. However, despite their critical role, N-S collaborations on datasets are not well understood. Science of science studies tend to rely on publications and patents to examine N-S collaboration patterns. To this end, the rise of global crises requiring N-S collaborations to produce and share data presents an urgent need to understand the prevalence, dynamics, and political economy of N-S collaborations on research datasets. In this paper, we employ a mixed methods case study research approach to analyze the frequency of and division of labor in N-S collaborations on datasets submitted to GenBank over 29 years (1992–2021). We find: (1) there is a low representation of N-S collaborations over the 29-year period. When they do occur, N-S collaborations display “burstiness” patterns, suggesting that N-S collaborations on datasets are formed and maintained reactively in the wake of global health crises such as infectious disease outbreaks; (2) The division of labor between datasets and publications is disproportionate to the global south in the early years, but becomes more overlapping after 2003. An exception in the case of countries with lower S&T capacity but high income, where these countries have a higher prevalence on datasets (e.g., United Arab Emirates). We qualitatively inspect a sample of N-S dataset collaborations to identify leadership patterns in dataset and publication authorship. The findings lead us to argue there is a need to include N-S dataset collaborations in measures of research outputs to nuance the current models and assessment tools of equity in N-S collaborations. The paper contributes to the SGDs objectives to develop data-driven metrics that can inform scientific collaborations on research datasets.

## 1. Introduction

Scientific collaborations between the global north and south (N-S collaborations) on research datasets are a critical component of addressing global crises and advancing public health research. Genomics research is one scientific area where N-S collaborations have proven crucial for advancing global, interdisciplinary research. For example, the SARS-CoV2 pandemic (COVID-19) and climate change relied on—and continue to depend upon—collaborations between scientists residing in the global north and south. N-S collaborations on genomics research data serve to facilitate the pooling of data, expertise, and resources to accelerate scientific breakthroughs (Crane, 1972; Bratt, 2022; Hemsley et al., 2022). In addition, N-S collaborations on datasets prevent the global spread of disease by collecting regionally-specific samples of disease variants and support vaccine development (Herzig Van Wees et al., 2019; Omotoso et al., 2022). The overall landscape of N-S collaboration on data in genetics and genomics research is increasingly globalized, and relies on international cooperation for dataset production and sharing (Costa et al., 2016; Lucas-Dominguez et al., 2021).

Despite these advances, there remains a “genomic data gap” (Omotoso et al., 2022). The genomic data gap refers to a lagging rate and volume of datasets submitted by specific regions to open research data repositories. Datasets are largely produced by high-income, western countries such as the United Kingdom, United States, and Canada (Cyranoski, 2021). For example, during the COVID-19 pandemic, African scientists deposited < 2% of the total SARS-CoV2 datasets deposited to Global Initiative on Sharing Avian Influenza Data (GISAID).

Part of the genomic data gap is the division of labor between the datasets and associated publications. That is, there may be a gap in who participates in the dataset production and to what extent they are also contributing to the publications. For example, African scientists contributed to Ebola by providing access to local populations but did not play a leading role in many of the collaborations, reflected by their low representation of African scientists as first authors on the resulting publications (Zhang et al., 2020). The division of labor in N-S scientific collaborations on datasets has important implications for innovation and equity outcomes. For instance, Xu et al. (2022) found that “flat” teams are associated with more innovative outcomes because less hierarchical teams meant that more of the collaborators participated in the core intellectual tasks.^1^ In large data-intensive teams, studies have found that a hierarchical organization of the technical labor and dataset preparation can be more efficient, but also leads to less generalizable outcomes due to the inaccessibility of the details of the data cleaning and analysis steps in the final manuscript reporting the results (Azoulay, 2019). Equity can be enhanced when the division of labor is peer-to-peer rather than hierarchical team structures because flatter teams are associated with increased knowledge sharing. For example, in N-S scientific collaborations with small, flat teams tend to more easily build research capacity for southern researchers and access to local populations for northern researchers (Wagner et al., 2015; Atkins et al., 2016).

Yet despite the critical role of the N-S division of labor datasets in shaping research outcomes, we still know little about N-S collaborations on datasets at scale and over time. For example, we lack empirical analyses of questions such as: How frequently have N-S collaborations on datasets occurred? What is the N-S division of labor on datasets? Have N-S collaborations on datasets increased with the maturity of global data sharing infrastructure? With a few exceptions (Chen et al., 2022; Omotoso et al., 2022), science of science studies tend to rely on publications and patents to examine N-S collaboration patterns. To this end, the increase in N-S collaborations on genomic datasets and the policy mandates to submit data to open research data repositories presents an urgent need to understand the longitudinal dynamics of the prevalence and division of labor in the N-S scientific collaborations on datasets.

It remains difficult to assess the United Nations (UN) Sustainable Development Goals (SDGs) around strengthening research capacity (Cash-Gibson et al., 2015; Lee et al., 2016) because we lack metrics using formal network terms (e.g., statistical, theory-driven). Without an understanding of N-S collaborations network structures and the division of labor on datasets the SDGs are undermined because we are left with a empirical gap in current models that inform science policy interventions, especially for the data-intensive sciences (e.g., genomics).

In this paper, we take a first step to characterize the prevalence and structure of international collaborations between scientists from countries in the global north and south on research datasets. We employ a mixed methods case study research (MMCSR) approach. We first conduct a bibliometric analysis using GenBank dataset metadata about collaboration over 29 year (1992–2021) and the World Bank country income classification and Science & Technology (S&T) Capacity Index (STCI) to analyze the frequency of and division of labor in N-S scientific collaborations on genomic research datasets. We then qualitatively examine a sample of collaboration clusters that include both scientists from the global north and south situate the quantitative results in context. Based on these findings, we discuss the implications for the use of the S&T capacity index and World Bank country income classifications for estimating collaborative equity and research capacity in the genomic context. The findings inform policy interventions that aim to strengthen research capacity in developing nations and monitor equity in international collaborations a UN Sustainable Development Goal (Lee et al., 2016). The longitudinal analysis of the division of labor on N-S collaboration on datasets is one of the first. As such, the study addresses the empirical gap as to the extent and distribution of work in scientific collaborations between scientists from the global north and south on research datasets.

This paper is organized as follows: We first provide a background literature review of the empirical landscape, with a focus on studies of N-S research collaborations in genomics on datasets and the division of labor on datasets. Next, we describe our research questions and the overarching study methodology (i.e., a “MMCSR” approach). We then detail the data sources used, data analysis techniques employed, and operational measures of the study. The findings follow and are structured by our two primary research questions. We offer a discussion of the findings and conclude with limitations and future work.

## 2. Background literature

### 2.1. Research datasets in genomics: global production and sharing

The “fourth paradigm” of science is an era characterized by more computation, collaboration, and data-intensive activities than prior scientific periods (Hey et al., 2009; Szalay and Blakeley, 2009). In genomics, research datasets are central to accelerating the vision of the fourth paradigm of science. A signpost of the fourth paradigm and centerpiece of dataset standardization and sharing is large-scale open research data repositories. Indeed, it is common practice for genomics researchers to typically deposit datasets into open research data repositories such as the National Center for Biotechnology Information (NCBI) GenBank (Benson et al., 2018), Protein Data Bank (RCSB PDB, 2023), and more generalized dataset archives like Dryad (Dryad Digital Repository, 2023). For instance, the Human Genome Project was enabled in large part by sharing datasets at a global scale through GenBank, and advances in addressing infectious disease research including COVID-19—owe their expediency to large open research databases.

A “genomic data gap” remains vividly apparent in both the sequencing technology capacity and the coverage of datasets in the African continent (Sirugo et al., 2019; Fatumo et al., 2022, p. 100). In the case of African genomic data production during COVID-19 pandemic, African scientists contributed < 2% of the total SARS-CoV2 sequence data due “lack of infrastructure and enabling environment for genomic studies, scarce or no funding and politics” (Omotoso et al., 2022). Moreover, Africans' genomic data only constitutes ~3% of the data used for genome-wide association studies 1.6% of genotype data in U.K. Biobank (Fatumo et al., 2022; Omotoso et al., 2022; Ramsay, 2022).

This genomic gap has begun to be addressed through capacity-building. The technological infrastructure for conducting sample collection and sequencing has been spearheaded by local organizations—e.g., the Nigerian 100k Genome Project (Fatumo et al., 2022)—and through collaboration with international partners. The next section reviews such collaborative efforts to partner between the global north and south to address the genomic data gap, and how N-S collaborations and the N-S division of labor on datasets.

### 2.2. Scientific collaboration between the global north and south on datasets

Global teams of scientists can accelerate health research by integrating multi-disciplinary expertise (e.g., epidemiology, transcriptomics) (Bietz and Lee, 2009). Collaboration at a worldwide scale also can ease the cost burden of specialized experiment and labor (Krueger, 1986; Chen et al., 2022). For these reasons, among others, it is advantageous for scientists affiliated with the global north and south to form partnerships. For instance, it is beneficial to develop N-S collaborations between researchers to study infectious disease. For instance, Zhang et al. (2020) showed that over four major global disease outbreaks that the United States (US) collaborated frequently on publications with several African countries on Ebola research (e.g., Sierra Leone, Guinea), the regions where Ebola primarily emerged to exchange expertise and access local populations. Likewise, regional expertise and distributed resources led to collaboration between China and the USA on SARS. These studies reveal the relationship of the regional outbreak and the scientific and technical capacity needed to address the disease burden.

However, N-S collaborations have been critiqued for their extractive and exploitative sometimes referred to as “helicopter science” approaches (e.g., Vanni et al., 2014; Atkins et al., 2016; Liverpool, 2021). Partnerships between N-S researchers of an extractive nature are not sustainable due to conflicts of interest and inequitable practices like unequal on data ownership, patent claims, and publication authorship (Omotoso et al., 2022). Studies specifically related to N-S collaborations in genomic suggest that solutions to extractive collaborations on genomics datasets must begin with “local research capacity building both in- and about Africa's health priorities.” They emphasize the importance of equitable divisions of labor for obtaining and sharing genomic data in Africa (Cash-Gibson et al., 2015; Omotoso et al., 2022). As Omotoso et al. (2022) emphasize: “Local researchers should consider forming a partnership with HIC collaborators who understand the context and needs of the African region, and assist in agenda development” (Omotoso et al., 2022).

Quantitative studies of science have begun to examine N-S scientific collaborations at scale, using large scale bibliographic metadata available through, e.g., OpenAlex, Web of Science, and Microsoft Academic Graph, to investigate the implications for research capacity strengthening for southern scientists. Studies have focused on equity concerns in N-S scientific collaborations, such as the tendency for N-S collaborations in research on marine biodiversity to increase the “collaboration capital” of western scientists (i.e., scientists from high income nations) and not that of scientists from low or low to middle income nations (Tolochko and Vadrot, 2021) and documented uneven N-S collaboration practices like “helicopter science” (Gazni et al., 2012; Haelewaters et al., 2021). Likewise, Gomez et al. (2022) found less citation attention goes to southern publications (Khanna et al., 2022). These inequities in collaboration and citation can exacerbate the already uneven investment in infectious disease outbreaks, a disease is carried largely by southern nations (Faure et al., 2021). Equity issues are likely to be amplified when a country's data infrastructure is immature.

A branch of the efforts to ameliorate the inequities is to focus on a flatter division of labor in N-S scientific teams. Early research has suggested that less hierarchical interactions between N-S researchers in a data-intensive genomic project can help to build scientific and technical (S&T) capacity. In the next section, we focus on these emerging studies of the structure and dynamics of N-S division of labor in data-intensive genomics research.

#### 2.2.1. Division of labor in north-south collaborations on datasets

The division of labor in data-intensive science has spanned from complex hierarchies to simple two-person teams. Large “big science” projects such as CERN, the Hubble Telescope, and the Apollo space program, have tended to embrace a hierarchical division of labor (Price, 1963; Collins, 2003; Turner, 2015). A hierarchical chain of command can facilitate the management of highly specialized, interlocking tasks of large-scale projects. For example, the Laser Interferometer Gravitational-Wave Observatory (LIGO) is a large-scale effort designed to conduct experiments and detect gravitational waves depends on a distributed expertise of a chain of labor (Collins, 2003). The LIGO scientists are organized into clusters and branches of smaller teams that answer to leadership among each tier. Like other space and astronomy research initiatives, LIGO is an international multi-disciplinary collaboration, including South American scientists. Other astronomy projects with scientists from countries with differing research capacity and economic status are those between European researchers and those residing in Chile (e.g., the ALMA radio telescope), Argentina (e.g., Q&U Bolometric Interferometer for Cosmology), and Brazil (e.g., the Longa Vista Observatory) (Collins, 2003). While these big science teams do contain “flatter” sub-groups, the structure of big science teams is still hierarchical, with the leadership residing a several steps removed from research “on the ground.” The division of labor is closely related to communication patterns, and the harmonization of the goals of all involved parties.

Unlike hierarchical teams, the tight-knit organization of flatter teams tends to contribute more directly to building and maintaining scientific and technical (S&T) human capital. S&T human capital is established and sustained through social ties and further access to S&T resources (Bozeman et al., 2001). In the context of N-S collaboration, flat team structures facilitate knowledge transfer and exchange because of the shared mental models that are developed during frequent interactions among all team members (Xu et al., 2022). For example, the short average path length of a flat network facilitates the rapid communication of information across the network, enabling the individuals at the periphery of the network to receive and potentially act on information with expedience. Also known as knowledge diffusion, which, in a crisis, is critical for developing solutions such as vaccines and for variant monitoring, the short average path means the community is often working with a friend-of-a-friend. Being close to your neighbor and your neighbor's neighbor has well-documented positive impacts such as mentorship, tacit knowledge transfer, collaborative trust, similar technology use (i.e., using the same software product) and network resilience (Haythornthwaite, 2002). Put another way, studies show flatter teams are associated with greater diffusion of information (Monteiro and Hanseth, 1996), in part because all the project scientists are involved in the core functions of the project such that direct communication and information exchange is facilitated.

A corollary to flatness found by researchers of interdisciplinary research (IDR) teams is that if the collaboration displays hierarchical structure, e.g., a distributed division of labor with few overlapping tasks that are central, there is less communication, and overall, fewer novel ideas generated (Xu et al., 2022). Their findings suggest that non-hierarchical division of labor in N-S collaboration networks can display features either constrain research capacity strengthening, such as hierarchical organization in the distribution of labor, intellectual elitism, and groupthink (Vlegels and Huisman, 2021). Flatter teams are associated with peer-to-peer exchange and can lead to research capacity strengthening (Bates et al., 2006; Haelewaters et al., 2021).

Despite these benefits, some studies also suggest that even in flatter teams, the power dynamics within a team can potentially have the opposite effect: constraining knowledge diffusion create power hierarchies (Newman, 2001; Rogers, 2010). For example, Tolochko and Vadrot (2021) measured the collaboration capital gained by western scientists vs. southern scientists after an international collaboration, finding western scientists more likely to benefit from the collaboration by gaining more collaborative ties and sustaining them longer. The study reflects how the structural properties of N-S collaboration networks correspond to an increment in research capacity. Tolochko and Vadrot (2021) suggested that the division of labor between the scientists may account for the discrepant outcomes in collaboration capital gained by western (northern) scientists.

Taken together, these studies highlight the benefits of N-S collaborations like increased research capacity and training, and shared resources. However, they also raise the question of whether flat, non-hierarchical teams are enough to support effective equitable outcomes. The paradox depends in part on the methods and metrics employed to measure S&T capacity and equity outcomes. For instance, the studies show that flat properties may not guarantee equity, though short-path lengths and clustering has been associated with strengthening peer-to-peer relationships (Armstrong et al., 2002). The metrics used are commonly based on publication data, even though the modern genomic research team has a broad distribution of labor. Because the studies are limited to publication data, they may miss the dataset labor central to contemporary genomics research. Including dataset authors in the analysis of the division of labor may allow us a clearer picture of the N-S collaborations. We lack longitudinal accounts of changes in the collaboration structures of N-S teams, too. If we can characterize the prevalence and division of labor on a key signpost of the global collaboration networks—data-intensive genomics collaborations on research datasets—we can develop clearer metrics and empirical insights into this central feature of the information age.

In this study, we systematically analyze the prevalence of N-S collaborations on datasets and the division of labor of N-S scientists on using the case of genomics research datasets submitted to GenBank (1992–2021). The following research questions guide our analysis:

**Research Question 1 (RQ1):** What is the prevalence of N-S collaborations on datasets?

This question guides our analysis of the frequency of collaborations on datasets occurring between scientists from countries with different income status and S&T capacity? All years (+ Plot the overall frequency of each n-wise collaboration) Yearly (1992–2021) + Plot the frequency of n-wise collaborations Mapping the countries with geographic visualization (overall, by year 1992–2021).

**Research Question 2 (RQ2)**: What is the division of labor in N-S collaborations on datasets?

We measure the division of labor using the overlap of authors between publications and datasets. We calculate the overlap of author names on the dataset and the publication. GenBank provides information about both the publication authors and the dataset authors. Here, we operate under the assumption that dataset authorship indicates the author is responsible for contributing to the dataset, as in the case of publication authorship. We provide further details on our methodological approach and materials in the following section.

## 3. Methodology

**Figure 1:**
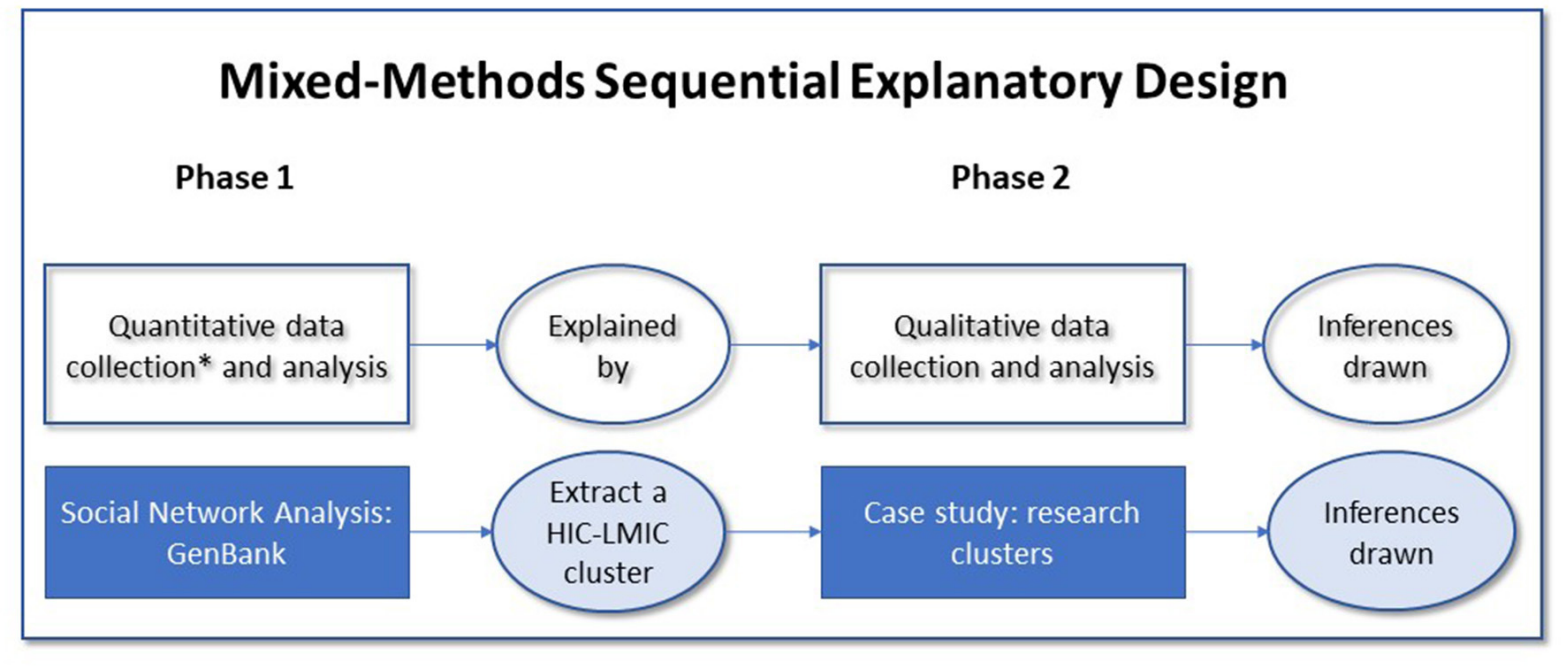
Study design: a mixed-methods sequential explanatory case study design in two phases.

In this study, we take an approach known as “mixed methods case study research” (MMCSR). The *mixed methods* approach integrates the multiple types and sources of data we need to address quantitative and qualitative aspects of our research questions (RQs). The prevalence of and longitudinal patterns characterizing N-S collaborations rely on *quantitative* methods (e.g., counting the frequency of N-S collaborations). To contextualize these quantitative patterns (e.g., the division of labor in N-S collaborations), we require both a quantitative characterization and qualitative techniques such as content analysis of documents produced in selected cases of N-S collaborations. The selected cases (i.e., the case study component of the “mixed methods case study research approach”) provide contextualized N-S collaborations of our specific cases for comparative analysis (Creswell and Poth, 2016). Within our MMCSR approach, we employ an “explanatory sequential design” in which we first perform quantitative data collection and analysis (phase 1), which is further explained by qualitative data collection and analysis (phase 2). In phase 1 we process and analyze the quantitative from the GenBank database. In phase 2, we purposefully sample the N-S collaborations and conduct a qualitative case study of the extracted teams (Figure 1).

The quantitative analysis is contextualized with a case study approach to examine the formation and development of representative collaborations between countries.

### 3.1. Data collection and processing

Data sources used for the primary quantitative analysis portion of this study from an ongoing project analyzing scientific collaboration networks (Qin et al., 2009; Costa et al., 2014, 2016; Bratt et al., 2017). The GenBank metadata from the project is contained in a relational database spanning 1984–2001, with a few earlier years (~0.1% 1900–1983). The data collected for the project is a fusion of multiple data sources: the metadata from the National Center for Biotechnology Informatics (NCBI) GenBank, the NCBI Taxonomy, the World Bank country income classification, and Scientific & Technical Capacity Index (STCI) data.

A GenBank annotation record consists of a section of metadata section and a section on molecular sequence data. These annotation records are available from the GenBank FTP server as compressed semi-structured text files. We downloaded all the annotation records from up until 2021 and extracted the metadata section from all annotation records, which were then parsed into a relational database (we excluded the genetic sequence data, which comprised 80% of the data volume). This process resulted in 227,905,057 annotation records, in which 44,480,172 publications were referenced.

To extract N-S collaborations from all GenBank records, we queried our database for all records with geographic information. The result was a year range of 1984–2021. Metadata attributes in the collection are title, journal, year, country, author names, institutional affiliation, and taxon data. The result includes a total of 13,467 references with 7,186 data submissions and 6,281 publications. There were 18,510 authors with 445,848 edges.

### 3.2. Measures: scientists' country affiliation and division of labor

We operationalize scientific collaboration using co-authorship on a paper or dataset. Co-authorship is a common measure of scientific collaboration in the bibliometrics literature (Beaver and Rosen, 2005; Costa et al., 2016; Wang and Barabási, 2021). The measure of dataset collaboration is less established, given its relative newness in studies of scientific collaboration on less “conventional” scientific products such as software and datasets (Li et al., 2016). Here, we extend the well-established logic of co-authorship on publications as a proxy measure for collaboration to datasets. That is, we infer that the co-contribution of two (or more) scientists on a GenBank dataset submission record is indicative of collaboration activity. However, if scientist X contributes to a publication but not its associated dataset, and scientist Y contributes to the dataset but not the associated publication, this poses a more difficult case to assume collaboration. Our study reported here analyzes the extent to which there is such an “overlap” of scientists on both the publication and dataset. It is out of the scope of this paper to develop a theoretical model of proxy measures of collaboration on datasets; future work can examine this direction.

Countries were classified per the 2019 World Bank's economic groups: low-income countries (LIC), low-to-middle-income (LMIC), upper-middle-income-countries (UMIC), and high-income-countries (HIC). The World Bank uses these income groups in the World Development Indicators database, which includes all the members of the World Bank (189 countries) and 28 other countries with >30,000 people as their population. The income groups' classifications change occasionally but are for the most part stable from year to year. We used the classification tables for each year of our data to classify countries to reflect the status of that country in that particular year and to track if they changed classification status in a later year. The income group categories are low, lower-middle, upper-middle, and high. To measure income, the World Bank uses the GNI per capita in U.S. dollars.

**Figure 2:**
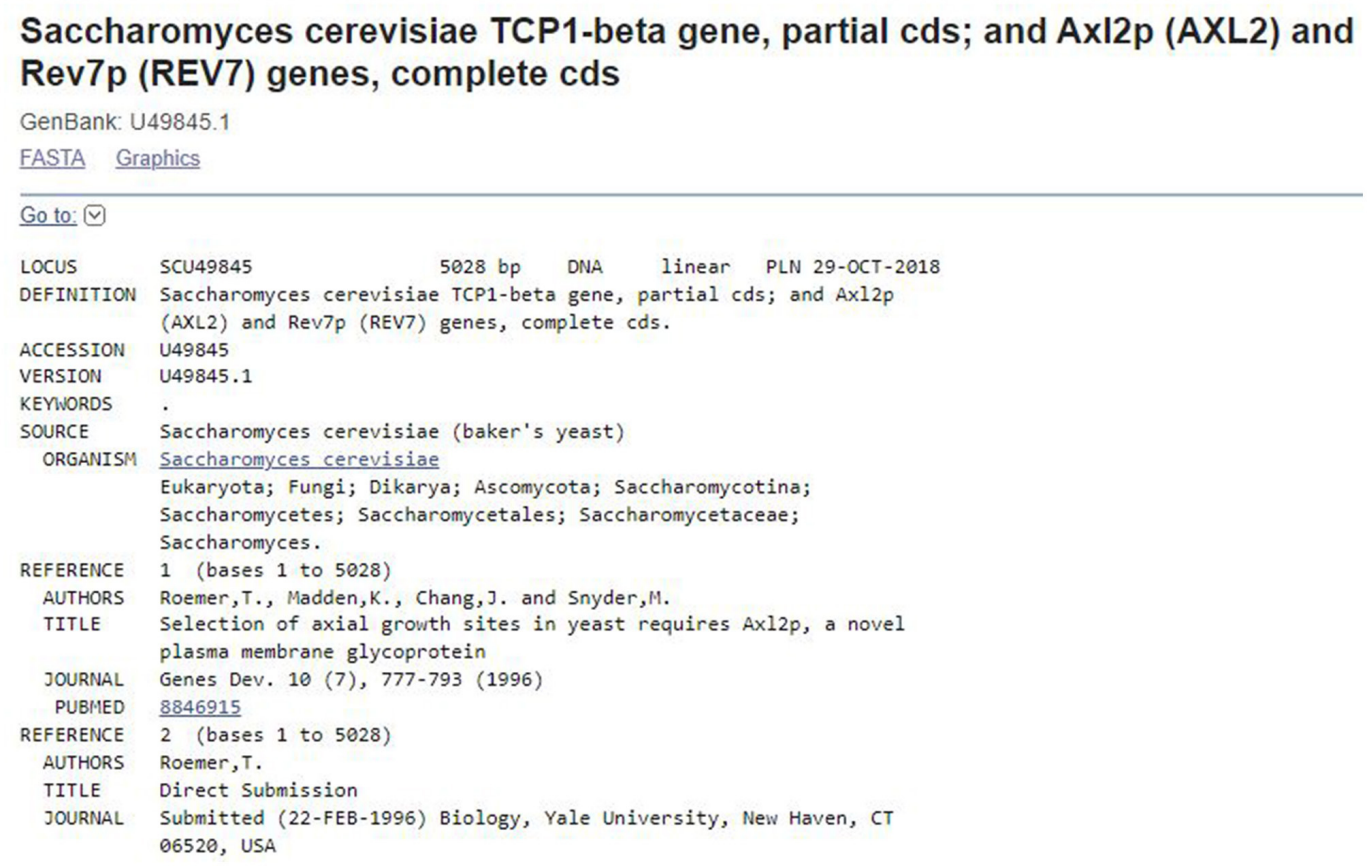
Sample GenBank annotation record.

Researchers have leveled compelling critiques of the use of the World Bank country income classifications (Wagner et al., 2001; Lencucha and Neupane, 2022), so we also incorporated an emerging measure specific to capacity, the Scientific and Technical (S&T) Capacity Index (STCI) developed by Wagner and Leydesdorff (2009) and can be accessed at: (National Science Capacity Index 2018-Unweighted, 2022) to triangulate classifying the countries, nuancing the N-S binary. We also classified countries according to an emerging measure, the Scientific and Technical (S&T) Capacity Index (STCI) (Wagner et al., 2015). The STCI accounts for economic, social, and technological features beyond the GNI or GDP to characterize a “nation's ability to carry out research” (Wagner et al., 2015). We operationalize the division of labor on datasets and publications with a measure of the ratio of authors on the dataset and publications of a single GenBank submission. A GenBank dataset submission is described by an annotation record (see Figure 2). The annotation record describes the dataset. The metadata in the annotation record includes the dataset authors, the date of the submission, and the related “references”—the publications most closely associated with the submitted dataset.

### 3.3. Case study analysis

Case studies provide in-depth contextual information specific to the situated phenomenon. The context of the two mixed income group cases were investigated using a qualitative case study approach of two components. These two components were extracted using network component analysis in R's iGraph package. We then identified the nodes in the cluster and extracted their affiliation metadata including title, co-authors, and outputs. Using the publications associated with a cluster, we then identified funding sources and selected policy outcomes, as well as media documents from the Centers for Disease Control (CDC) articles, events reported in media documents (i.e., news articles) associated with mixed-income group statistics. We also used institutional documents including calls for funding proposals, institutional initiatives, published policy outcomes associated with clusters. To measure research capacity, we conducted qualitative deductive analysis of the documents, identifying the places where new collaborations were formed, to reflect collaboration capacity (as our proxy measures for research capacity strengthening).

## 4. Findings

In this section, we report the findings of the analysis, guided by our two research questions: **RQ1:** What is the frequency of N-S collaborations on datasets submitted to GenBank? **RQ2:** What is the division of labor in N-S collaborations on datasets submitted to GenBank?

### 4.1. Frequency of N-S collaborations (1992–2021)

We analyzed the frequency of scientific collaborations involving countries with differing World Bank country income classifications and differing S&T Capacity Index (STCI). The use of these classification systems allowed us to nuance the N-S binary, representing the countries on a more accurate, granular level. In the analysis of the frequency of the N-S collaborations, we first conducted descriptive statistics of all scientists who submitted datasets to GenBank. A total of 105 countries have submitted datasets to GenBank (1992–2021). Note that scientists can be affiliated with multiple countries, but that this in a minority of cases (12% percent of scientists in our sample).

**Figure 3:**
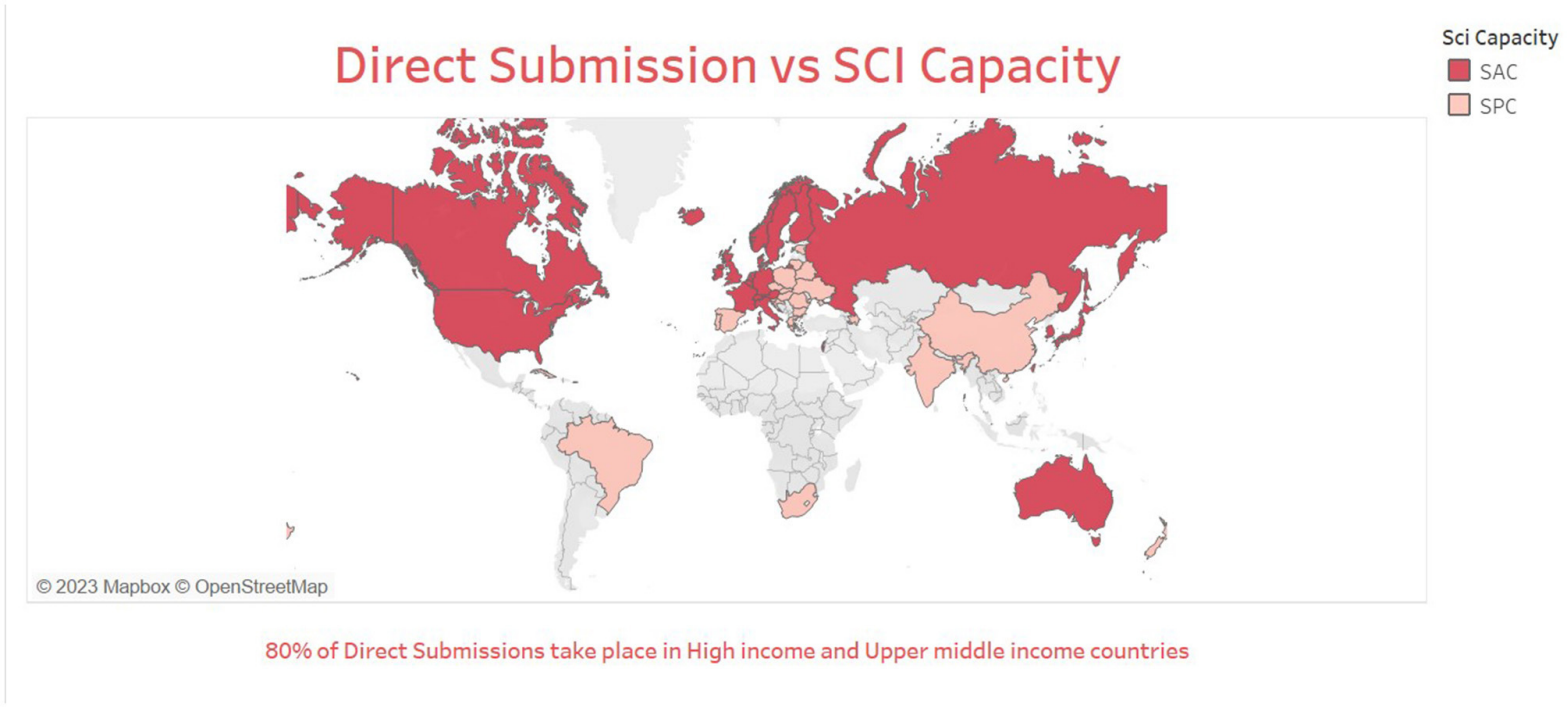
Map of world with N-S collaborations.

From 1992–2021, most of the dataset collaborations are constituted of scientists from high-income countries (HIC) like the United States, France, and Canada. The analysis conducted on North-South collaboration aimed to explore the distribution of collaborations among different countries. We found that the majority of direct submissions (**95%**) came from scientifically advanced and progressing countries, and that **71.71%** of these countries classified as high-income nations according to the World Bank country income classification. Among these, the most frequent international collaborations on datasets were among scientifically advanced countries (see Figure 3).

The most frequent dataset submitters who were from countries with lagging capacity are Thailand, Malaysia, and Algeria. The scientists from these nations collaborated with the UK and France. Over time, the N-S collaborations on datasets occurred a total of 11,324 times (i.e., ~11 k dataset submissions), which increased over the span of 29 years. When the whole network data was filtered for only components with N-S researchers the network size decreases sharply. The team size of these countries is larger on average than the collaborations among advanced nations.

**Figure 4:**
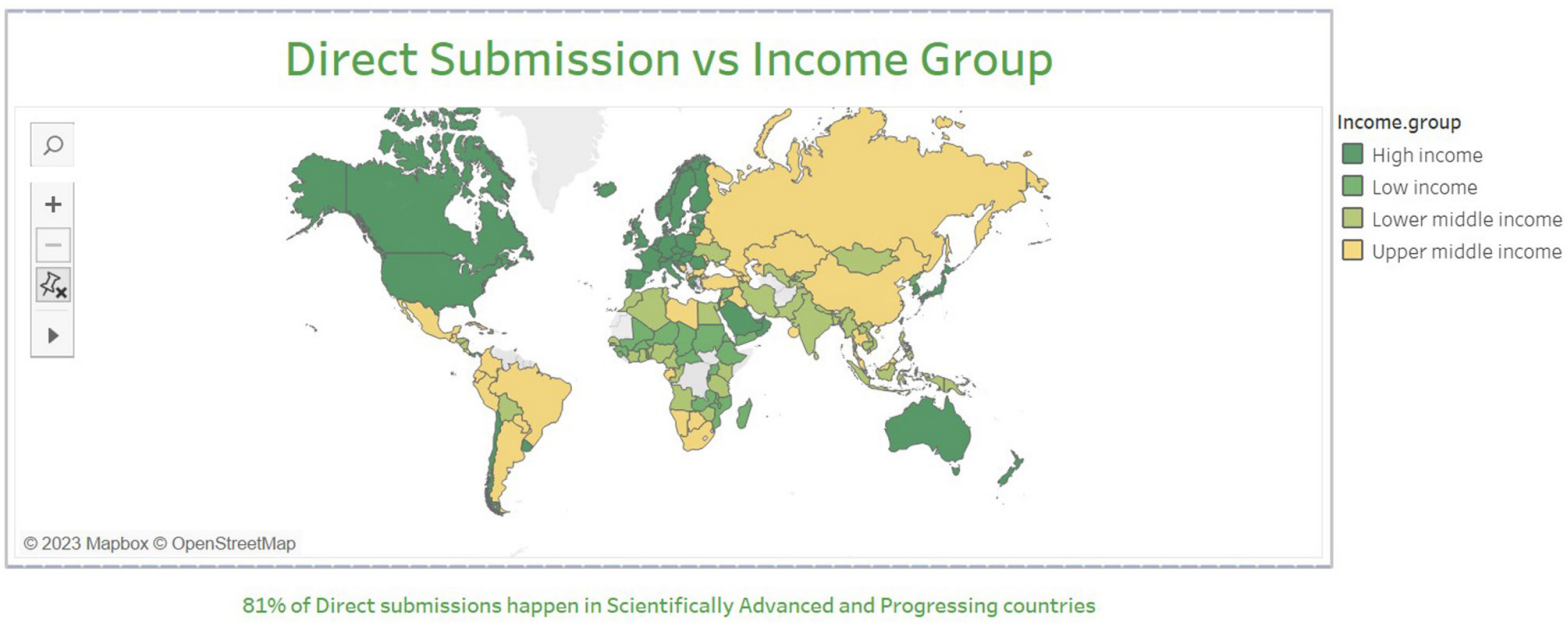
Team size over time.

Figure 4 shows the appearance of larger N-S teams over time. The incidence of larger connected components with researchers from well-resourced countries suggests the organization of larger efforts by larger institutions, given the size of the component is health outbreak events requiring HIC collaborative efforts. We find that when there are collaborations, there is “burstiness”—collaborations that occurs because of work on infectious disease outbreaks. For example, the Ebola virus led to collaborations between researchers from Ethiopia, Jordan, and the United Stated. The topics on which N-S collaborations on datasets tend to occur are related to infectious disease outbreaks, and their timing of publishing tends to coincide with the disease outbreak.

### 4.2. Division of labor on datasets and publications

As one of the objectives of this study, we try to answer the question of how many authors part of the dataset submissions to GenBank were also part of the publications associated with the very same dataset submissions. This overlap of authors between dataset submissions and publications is what we call “author overlap”. We performed an analysis of the fraction of author overlap, the average and median dataset submissions and publications team size overall and across the years from 1992 to 2021 (Table 1).

**Table 1:** Division of labor statistics by year.

**overlapping_count**	**non_overlapping_count**	**total_count**	**fraction_overlap**	**pub_count**	**sub_count**	**avg_pub_team_size**	**avg_sub_team_size**	**median_pub_team_size**	**median_sub_team_size**	**only_sub_count**	**only_pub_count**
2,775,677	3,128,059	5,903,736	0.47016	4,540,349	3,713,755	5.2	4.2	4	3	938,078	1,764,672
4,235	18,180	22,415	0.18894	21,036	5,281	4.4	1.1	4	1	1,046	16,801
7,190	27,470	34,660	0.20744	32,536	8,822	4.4	1.2	4	1	1,632	25,346
9,574	40,207	49,781	0.19232	47,303	11,406	4.5	1.0	4	1	1,832	37,729
16,771	46,814	63,585	0.26376	59,250	19,765	4.6	1.5	4	1	2,994	42,479
29,241	49,886	79,127	0.36955	73,139	32,733	4.7	2.1	4	1	3,492	43,898
42,895	60,929	103,824	0.41315	92,440	49,775	5.0	2.7	4	2	6,880	49,545
55,802	77,711	133,513	0.41795	110,948	71,093	5.0	3.2	4	2	15,291	55,146
69,779	103,549	173,328	0.40258	133,983	97,505	5.4	3.9	4	2	27,726	64,204
79,032	123,033	202,065	0.39112	148,414	117,828	5.5	4.4	4	3	38,796	69,382
75,834	124,088	199,922	0.37932	136,914	122,894	5.2	4.7	4	3	47,060	61,080
88,547	297,258	385,805	0.22951	157,581	270,717	5.3	9.1	4	3	182,170	69,034
86,359	234,364	320,723	0.26926	153,930	218,271	5.0	7.2	4	3	131,912	67,571
90,832	258,266	349,098	0.26019	160,597	238,949	5.3	7.9	4	3	148,117	69,765
94,703	294,220	388,923	0.2435	165,374	271,746	5.0	8.4	4	3	177,043	70,671
101,073	144,995	246,068	0.41075	172,431	155,407	5.3	4.8	4	3	54,334	71,358
106,551	112,963	219,514	0.48539	176,495	135,590	5.3	4.0	4	3	29,039	69,944
111,997	94,079	206,076	0.54347	175,043	131,027	5.0	3.8	4	3	19,030	63,046
123,900	90,720	214,620	0.5773	191,944	134,239	5.4	3.8	4	3	10,339	68,044
136,542	92,137	228,679	0.59709	203,903	147,152	5.4	3.9	4	3	10,610	67,361
135,243	89,247	224,490	0.60245	204,907	141,780	5.4	3.7	4	3	6,537	69,664
142,412	87,693	230,105	0.6189	211,716	147,311	5.3	3.7	4	3	4,899	69,304
144,122	91,226	235,348	0.61238	217,430	148,988	5.3	3.7	4	3	4,866	73,308
149,352	87,261	236,613	0.63121	219,081	153,753	5.2	3.6	4	3	4,401	69,729
149,011	82,915	231,926	0.64249	215,770	152,360	5.0	3.6	4	3	3,349	66,759
150,452	79,644	230,096	0.65387	214,921	152,948	5.0	3.6	4	3	2,496	64,469
148,470	71,894	220,364	0.67375	207,556	149,210	5.0	3.6	4	3	740	59,086
143,746	73,279	217,025	0.66235	204,372	144,673	5.0	3.5	4	3	927	60,626
131,544	71,095	202,639	0.64915	191,123	132,428	5.0	3.4	4	3	884	59,579
118,292	60,018	178,310	0.66341	169,433	116,465	5.4	3.7	4	3	−1827	51,141
22,516	5,949	28,465	0.79101	27,505	21,490	5.0	3.9	3	3	−1026	4,989

**Figure 5:**
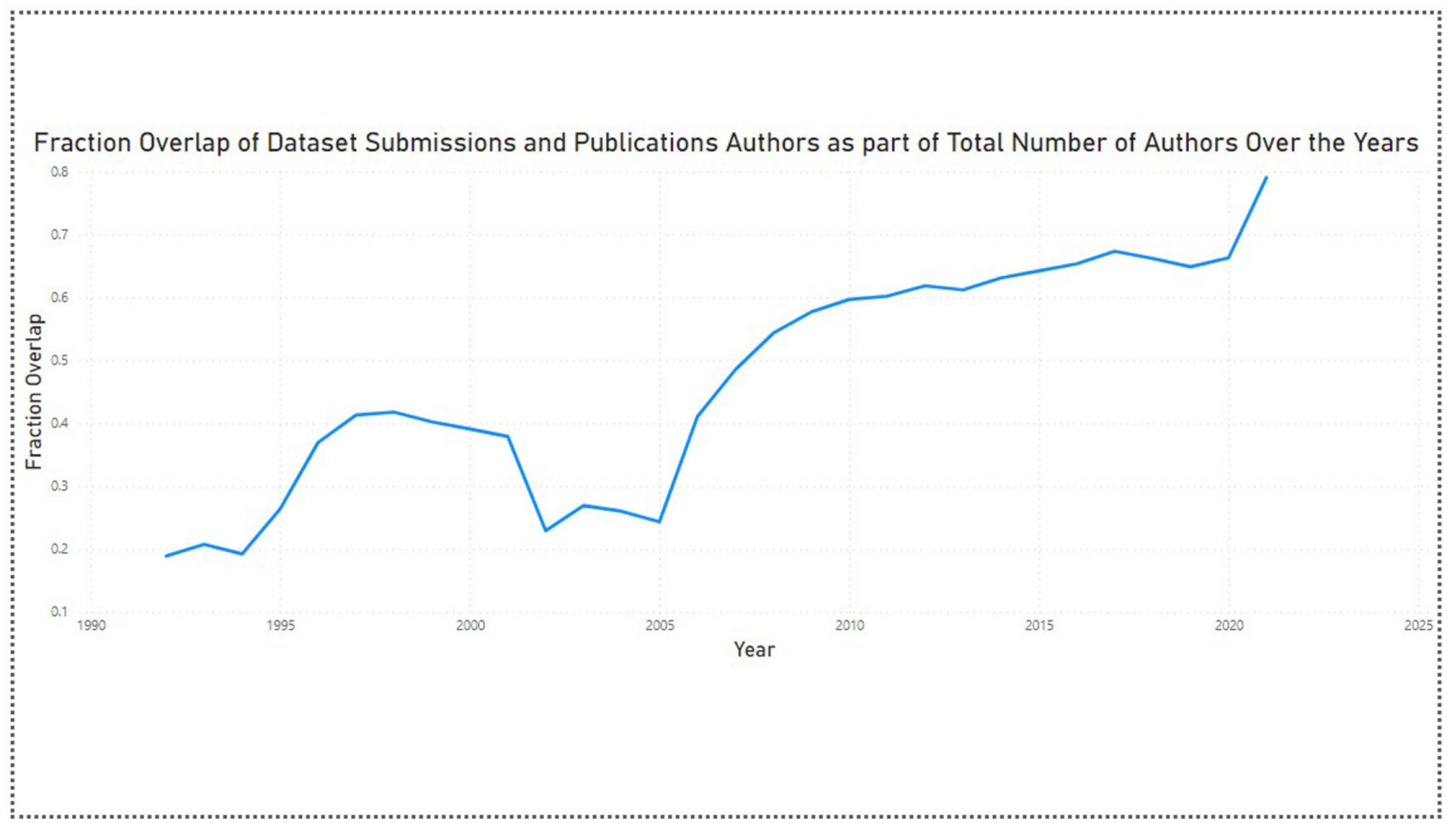
Author overlap ratio 1992–2021.

The analysis shows us that the overall fraction overlap has a value of 0.47, which implies that 47% of the total number of authors contributed to the datasets as well as the publications. This fraction overlap value increased from 0.18 (18%) in 1992 to 0.79 (79%) in 2021. This indicates more authors started contributing toward the datasets across the years. This conclusion has also been supported by the fact that the average dataset submission team size increased from 1.1 in 1992 to 3.9 in 2021 (Figure 5).

**Figure 6:**
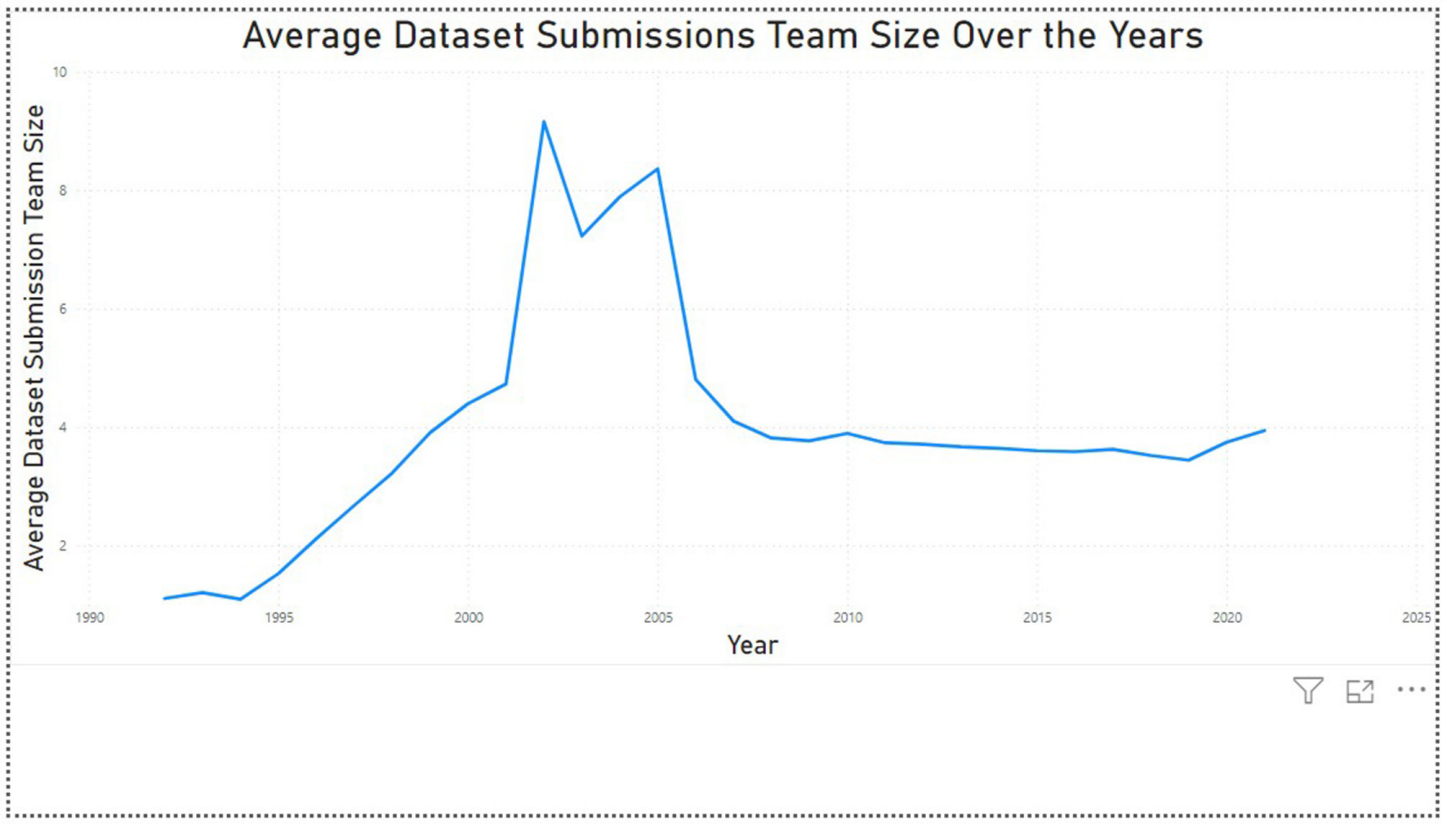
Average dataset team size (1992–2021).

There is no major difference in the mean (average) and median values of the dataset submissions and publications team size over the years (Figure 6). This indicates that there are no outliers present in the dataset. For further analysis, we can incorporate the countries the authors are associated with to analyze the trends in the collaborations between the global North and South countries, and which contribution (dataset submission or publication) these countries are associated with.

We selected two teams from this sample to examine two research groups with mixed income (HIC-LMIC) researchers. The two components identified are international collaborative initiatives were connected to the Harvard Botswana Partnership (HBP) and the Brown University/Tufts University AIDs International Training and Research Program (AITRP). We searched the literature describing and produced by the institutions engaged in the collaboration (e.g., scholarly, institutional, and media reports). The case studies of these two research groups serve as a deep dive into mixing patterns over time to provide a richer contextualization of cases. We selected these two components as candidate cases because they represented research groups comprised of mixing patterns. The two components were consistently producing publications and dataset, providing cases of successful HIC-LMIC research group collaborations. We visualized the networks and colored the nodes according to the income group as an initial exploration of existing mixed HIC-LMIC components.

After visual examination, the clusters with mixed HIC-LMIC components were computationally extracted and the metadata for publications, datasets, and additional contextual information were queried. Based on these metadata, we conducted a document search on Google Scholar and the researchers' professional websites, and media and web content (e.g., funding documents, press releases). Based on the earlier analysis of component size distributions, we identified two mixed-income components of representative size which had a sustained presence in the network (1992–2018).

The first component-case study identified was associated with four countries: USA, Thailand, Cambodia, and France. The second component-case study was associated with the Harvard Botswana Partnership (HBP). While France and the USA have consistently (over the last 100 years) been classified by the World Bank as high-income countries, Thailand and Cambodia have historically switched lending groups and income group classifications since the 1980s, but slowly have climbed the ranks in both respects. Thailand has become a global leader against HIV. Thailand has undergone socioeconomic development in the last century, “moving from a low-income to upper-middle-income country in less than a generation” (Siraprapasiri et al., 2016). Beginning in the 1970s, the Royal Thai Government invested in health infrastructure showing a “significant and sustained” commitment to health (Siraprapasiri et al., 2016) by building health care facilities and establishing a health care workforce.

The second case study component reflects a research-capacity building effort initiated by the partnership called the Botswana-Harvard Partnership (BHP). The BHP recently celebrated a 20-year anniversary, indicating the collaboration was a concerted capacity-building effort rather than a “serendipitous” collaborative partnership. Botswana, like Thailand and Cambodia, has shifted income classification and lending group categories. It is currently an upper middle-income country, as of FY 1995. While it has gained independence, it has lost funding support from global health agencies because of its reclassification as an upper middle country. Despite its economic growth, Botswana remains one of the most severely impacted countries of HIV. It has established a provision for universal free antiretroviral treatment.

## 5. Discussion

Systematic analyses of the dataset authorship are nascent. Our findings indicate a data gap between scientists residing in high income, scientifically advanced nations, providing insight into the division of labor on datasets. However, the precise meaning of dataset authorship in GenBank records is ambiguous. Dataset authorship could indicate an author is the owner, creator, and/or submitter of the dataset. For example, found the person, e.g., a postdoctoral fellow, who submitted the dataset to GenBank. The early years of dataset submission to GenBank lacked clear guidelines about who should appear as a “dataset author.”

Further, the metrics for measuring S&T research capacity in dataset terms are likewise in their early stages. Just as promotion and tenure (P&T) use of unconventional metrics such as datasets and software remain largely publication-centric, so too have indicators for the UN sustainable development goals (SDGs). In this analysis, we saw how the use of World Bank income status vs. S&T Research capacity might influence our interpretation of collaboration to inform metrics and indicators of research capacity. There are some countries such as the United Arab Emirates where they are scientifically lagging, but a high-income nation. As such, using classifications of countries in our analyses of metrics is important. Likewise, we can ask: How does the N-S Language help and hinder us? What are the strengths and limitations of using GenBank data to develop indicators of dataset production and collaborations? Our findings that the use of the S&T human capital classification allowed us to see the nations in terms of their advanced scientific capacity, whereas the World Bank did not specify the capacity of a scientists' country in those terms).

North-South Collaboration on Datasets and Research Capacity We found that collaboration between mixed income groups and S&T capacity groups were sporadic and infrequent, as indicated by the low prevalence in the network. At the same time, other bibliometric analyses have complicated the story of N-S scientific collaboration. First, we cannot depend on measures of the presence and frequency of collaboration between N-S as an indicator of capacity-building. As Wagner et al. (2001) point out, just because there is N-S collaboration does not guarantee that capacity-building or strengthening occurred between the global north and south collaborators. In fact, the collaboration may have been exploitative or initiated to address a topic of interest to the high-income partner, but of little local benefit. Second, distribution location of high-income countries as the core of the network can be indicative of overrepresentation of HIC.

However, we also found that the collaborations between the mixed income groups have significantly larger components. These results suggest mixed income group collaborations are a result of intentional institutional efforts to build partnerships, such as those seen in the case studies. The presence of large components suggests a level of network cohesion, where the long-term relationships can lead to the diffusion of knowledge. The Thailand-Cambodia-France-USA partnership showed a growth in the health infrastructure associated with increased scientific collaboration in genomics. Likewise, the Botswana-Harvard Partnership (BHP) reflected a committed effort over multiple decades to develop programs and research capacity, coincident with public health outbreaks. The partnerships can result in increased research capacity as well as research breakthroughs, evidenced by the phasing out of the antiretroviral stavudine in Thailand.

### 5.1. Division of labor metrics to assess collaborative equity

Using quantitative studies of science for decision-making about strategies for supporting equitable collaboration between N-S has many benefits. First, aggregate patterns at the macro- and meso-levels of scientific collaboration networks reveal broad trends and the impacts of policy intervention at the network level, showing the outcomes or ripple effects of policy decisions on collaboration dynamics. Such at-scale studies enable us to quantify the effect size of policy interventions. They are becoming more feasible and less “niche” due to the rise of bibliometric data from databases like OpenAlex (Priem et al., 2022), Microsoft Academic Graph (MAG), and Semantic Scholar (Fortunato et al., 2018). However, as in the case of this study, open research data repositories such as NCBI's GenBank, are difficult to access because of their technical barriers to collecting, cleaning, storing, and accessing the data (Qin et al., 2009).

Since “north-south” is conceptually problematic (Wagner et al., 2001), we can also look to other indices to measure capacity. A methodological component of the analysis is the selection of a schema to classify countries according to their different economic, social, and technological features relevant to the study. This classification step is often overlooked, or at least unreported in analyses of quantitative analyses of scientific activity related to the “global north” and “global south.” The selection of classification of countries to group them according to their differential income and S&T capacities. Up to this point in this paper, we have not problematized the N-S binary, referring to countries as belonging to “global North” or “global South.” The N-S divide is a relatively common, colloquial way to refer to countries with historically discrepant economies. But upon closer inspection, the terms global north and global south do not accurately characterize countries, or the scientists from those countries. Rather, the N-S classification is a coarse-grained binary complicated once we interrogate the classification parameters.

Following Lucy Suchman's critique of *Artificial Intelligence* (AI) as a “floating signifier” (Suchman, 2020, 2022), that is, an abstract term to describe a broad phenomenon in ways that are “slippery,” and ultimately “escapes concrete definition as a referent” (Suchman, 2022), we argue the N-S classification is a floating signifier. Here, it is unclear what is the actual referent of a country in the “global North” (i.e., a country, say, Ethiopia or the United Kingdom)? Previous studies have used the World Bank country income classification to represent countries' economies along relative income dimensions measured by Gross National Product (GNP) (e.g., high income countries vs. upper-middle-income countries). Income statuses change on a yearly basis. We reflect further on the strengths and limitations of developing metrics for assessing N-S collaborative equity and research capacity, focusing on how to employ these classification schemas for developing quantitative assessment tools of research capacity and collaborative equity at scale: the S&T Capacity Index (STCI) and the World Bank country income classification.

### 5.2. Limitations

There are well-documented limitations to quantitative approaches using bibliometric trace data for studying equity in scientific collaborations. In general, co-authorship is not neither a comprehensive nor consistent proxy for the nuanced relationships and dynamic social interactions unfolding in scientific collaborations, nor the political or socioeconomic landscape in which scientists collaborate. Second, dataset co-authorship norms are not well studied in the science of science or social studies of science (e.g., in STS), leading us to assume that dataset co-authorship is indicative of contributions to the dataset. Third, quantitative modeling using archival data provides a delayed view. Publication co-authorship as a proxy for collaborations represents the finished product of the collaboration not a real-time representation (Glänzel and Schubert, 2005; Bratt et al., 2017).

Real-time bibliometrics is a work-in-progress by quantitative studies of science scholars (e.g., Hook et al., 2021). The World Bank country income classification dataset is for only 2019; however, there are yearly datasets available to assign the country label (e.g., high income, low income) according to its status classification every year. If there are changes from year to year as was the case for several countries during COVID-19, the category may change influencing the frequency counts of the country that year. Therefore, it is a limitation of the study. Future research will analyze the yearly classifications by using the yearly classifications of the World Bank data. We assume that if countries change categories, it is to progressively increase their status (e.g., in the case of Russia, India, and Thailand who went from low income to upper middle or high income). Future research will add the yearly granularity to measure the impacts of collaboration dynamics on the change in a countries' income status. Future studies can also build on this work by investigating the relationship of team size and the author order on the increment of research capacity.

## 6. Conclusion

In this study, we systematically analyzed the frequency and division of labor on N-S collaborations on datasets. This analysis of the prevalence and structure of collaboration on datasets is one of the first of its kind, to our knowledge, because it offers a quantitative longitudinal first approximation of the extent and structure of scientific collaborations between scientists from the global north and south on research datasets. The mixed-methods case study research approach provided a longitudinal empirical analysis of the frequency of N-S collaborations and their co-authorship dynamics over time, science policy can better support the N-S collaborations in the data-intensive sciences. We found the division of labor on datasets has increased in its overlap, suggesting that teams in genomics may be becoming more “flat,” with scientists sharing core tasks on both writing and data production. By understanding the collaboration network structures and dynamics on datasets, we can better design interventions to support data-intensive collaborations in future global health crises. Future research can analyze the division of labor on publication vs. datasets.

## Data availability statement

The datasets presented in this study can be found in online repositories. The names of the repository/repositories and accession number(s) can be found below: https://github.com/jnqin86/collabnetwork.

## Author contributions

SB: conceptualization (lead), formal analysis (lead), writing-original draft (lead), writing-review and editing (lead), supervision (lead), project administration (lead), data visualization (equal). ML and AN: data visualization (equal), writing-original draft, formal analysis (equal).

## Footnotes

^1^Bratt, S., Gomez, C. Lee, J., Langalia, M, Nanoti, A., and Leahey, E. [Under review]. Division of Labor on Scientific Datasets [Unpublished manuscript]. University of Arizona, School of Information.
